# Cement leakage and complication of liposarcoma spinal metastasis during vertebral augmentation procedure: a case report

**DOI:** 10.1186/s13256-016-0828-4

**Published:** 2016-02-24

**Authors:** Aykut Akpinar, Necati Ucler, Cem Seyho Yucetas, Uzay Erdogan, Mehmet Davut Ucar

**Affiliations:** 1 Department of Neurosurgery, Adiyaman University Education and Research Hospital, Adiyaman, Turkey; 2 Department of Neurosurgery, School of Medicine, Adiyaman University, Adiyaman, 02200 Turkey

**Keywords:** Cement leakage, Myxoid round cell liposarcoma, Radiofrequency tumor ablation, Spinal metastasis, Vertebral augmentation

## Abstract

**Background:**

Liposarcoma is a malignant tumor of soft tissue. Myxoid/round cell liposarcoma has a tendency to spread to extrapulmonary sites but the spine is an unusual location even for metastasis. Metastatic bone tumors in the spine are painful. The vertebral body augmentation procedures for treating painful metastatic spinal lesions are minimally invasive and are good alternatives to open surgery.

**Case presentation:**

A 41-year-old Turkish man was treated with radiofrequency tumor ablation and percutaneous vertebral augmentation for spinal metastasis. Asymptomatic perivertebral and segmental veins' cement leakage was detected on perioperative X-ray radiograms; at the follow-up computed tomography scan, no further migration of any cement material was seen, and his postoperative course was uneventful.

**Conclusions:**

The risk of cement leakage and embolism is increased with the treatment of some malignant lesions. The frequency of local leakage of bone cement is relatively high. Patients undergoing percutaneous vertebral augmentation of malignant spinal metastases need close monitoring. There is no agreement on the treatment strategy.

## Background

Liposarcoma is one of the most common histologic types of soft tissue sarcoma [1]. The frequency of myxoid/round cell liposarcoma (MRCL) metastasis to bone is not clear [2], but the incidence in one series was reported to be 17 % [1, 2]. The round cells (>5 %) are considered high grade [1]. The patients were followed up clinically with computed tomography (CT) or chest X-ray surveillance for metastasis. Magnetic resonance imaging (MRI) provides the most sensitive technique for the diagnosis of bone metastasis in MRCL [2].

Spine metastasis can be debilitating, and have a significant impact on patients' quality of life. The treatment regimen for spinal metastasis is generally palliative and consists of a combination of medical therapies (steroids, pain medication, chemotherapy, radiation therapy, and surgery). Radiofrequency thermal ablation (Rf-TA) and vertebral augmentation procedures (VAPs) have been shown to be effective in metastatic bone lesions [3, 4]. Our patient received vertebral augmentation with high-viscosity cement after use of Rf-TA. Although relatively safe, the procedure is not without risks.

## Case presentation

**Fig. 1:**
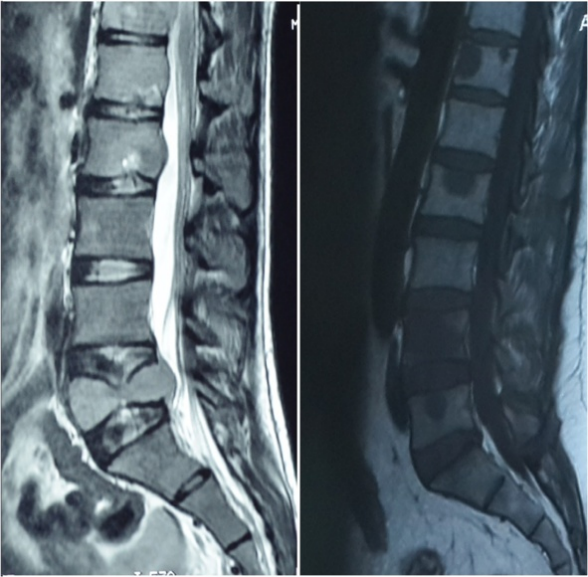
Spinal preoperative T1-weighted and T2-weighted magnetic resonance imaging revealed multilevel vertebral body edema

We describe the case of a 41-year-old Turkish man. Eight months before he was admitted to our clinic, a subcutaneous tissue mass was excised with a marginal excision from his right thigh; it was liposarcoma. He then received chemotherapy and radiotherapy with a good response but the liposarcoma had since progressed. He was admitted to our hospital, presenting with back pain with radiation and with no neurological deficits. He had a 3-month history of increased back pain, and he did not recall any trauma. A skeletal survey showed no lytic lesions, and a CT scan also showed no evidence of tumors in his spine. A spinal MRI revealed multilevel vertebral body edema (a low signal was seen within the body of his lumbar and thoracic vertebrae, on T1 corresponding to high signal on T2, in keeping with an interosseous metastasis; Fig. 1). A CT scan of his chest showed lung metastasis. There was no cord compression. After he and his family gave us written informed consent, Rf-TA and VAP were performed.

### Surgical technique

**Fig. 2:**
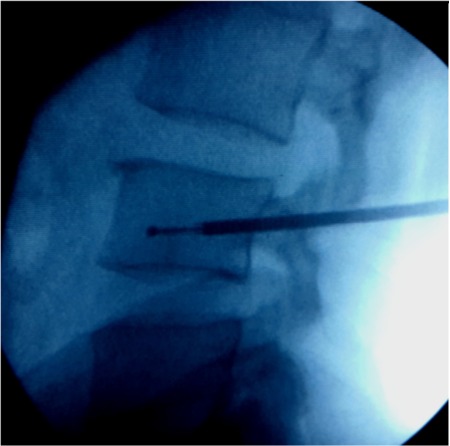
The procedure was performed under fluoroscopic guidance, and thermal ablation (50 to 80 °C, 10 minutes) was made

**Fig. 3:**
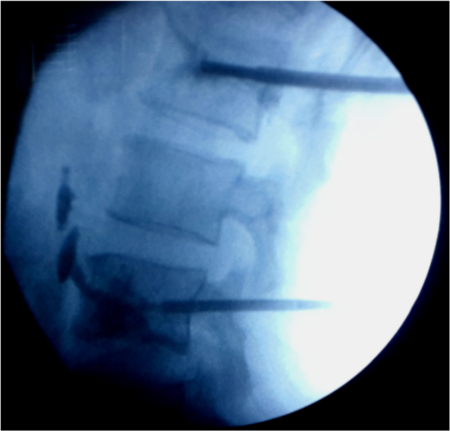
The fluoroscopic image showing cement leakage

Under spinal anesthesia in a prone position, this procedure was also performed with bi-plane fluoroscopy through a transpedicular approach by placement of working cannulas unilaterally. Through the working cannula, a drill and curette were used to create a tract and a cavity at the center of L2 to L4 vertebral bodies. The procedure was performed under fluoroscopic guidance and Rf-TA (50 to 80 °C, 10 minutes; Fig. 2) was made. We then injected high viscosity bone cement at low pressure into the ablated tumor bed. The bone cement injection was monitored with continuous fluoroscopy. When leakage was noted, the application was stopped (Fig. 3). In total, 4 ml cement had been released. Postoperative serum chemistries, arterial blood gas, and cardiac enzymes were normal and his postoperative course was uneventful. Cement leakage was observed in the perivertebral soft tissue, perivertebral venous system, and segmental vein.

**Fig. 4:**
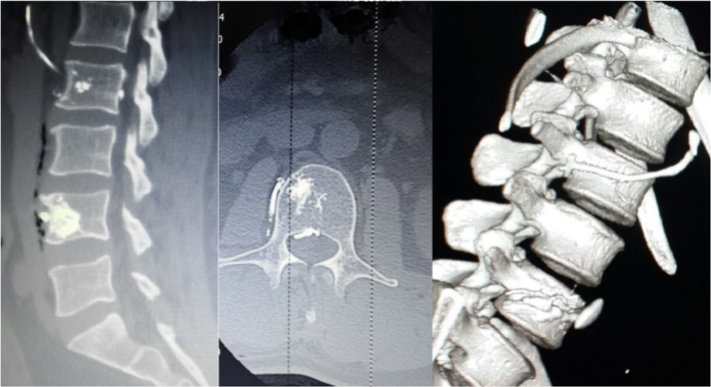
A computed tomography scan showed the characteristic appearance of cement leakage at the level of augmented vertebrae in the perivertebral venous system, segmental vein, epidural space, and soft tissue

Although our patient remained asymptomatic, he had X-rays and CT scans prior to discharge on postoperative day 7. A repeat CT scan showed the characteristic appearance of cement leakage at the level of augmented vertebrae in the perivertebral venous system, segmental vein, epidural space, and soft tissue (Fig. 4). A follow-up CT scan showed no further migration of any cement material.

## Discussion

The most common subtype of soft tissue sarcoma is liposarcoma, which constitutes 9.8 to 18 % of cases [5]. The peak incidence of MRCL (male predominant) is in the fourth and fifth decades [5]. MRCL in particular tends to spread to other soft tissue sites, including the retroperitoneum, thorax, opposite extremity, and other soft tissue sites, before metastasis to lungs. The skeletal metastasis prevalence is unclear [6].

Early detection of metastasis can affect the quality of life of patients. Initial staging and follow-up studies should include chest CT, bone scanning, abdominal and pelvic CT, and MRI. For bone metastasis, MRI is the investigation of choice [2]. Although intensive multimodality therapy has prolonged the survival of patients with musculoskeletal sarcomas, the prognosis of patients with metastatic disease is still poor. The reason for this is the high incidence of extrapulmonary metastases.

Rf-TA and VAP have been shown to be effective in metastatic bone lesions [3, 4]. Despite the high rate of success with VAPs, perioperative and postoperative complications are encountered mostly from cement leakage into the surrounding tissues and systemic circulation. The frequency of local leakage of bone cement is high (63 to 81 %) [7]. Moreover, the rate of perivertebral venous leakage is 88 % [7]. Discal leakage (34 %) with consequent pulmonary cement embolism varies from 4.6 to 6.8 % (up to 26 % in radiologic studies) [8, 9]. Cement leakage into the paravertebral veins, and pulmonary, cerebral, and cardiac embolism are severe complications [7–10]. As a result of bone cement leakage into venous channels, lethal conditions such as pulmonary embolism can occur, with rates ranging from 0.6 to 0.01 % [11]. Cement leakage into the paravertebral soft tissue (6 to 52 %) and intervertebral disc space, increasing pain, hypotension, and new fracture risk are mild complications [12]. Infection, misplaced needle, and epidural and foraminal space cement leakage (the prevalence may be as high as 40 %) are moderate complications [12, 13]. The rates of neurological complications with VAPs were between 0.6 and 0.03 % [11, 13].

Bone cement leakage complications occur more often when treating patients with metastatic disease (<10 %) than those with osteoporosis (1 to 2 %) or spinal angiomas (2 to 3 %). The higher risk of extravasation in patients with spinal malignancy is because of the cortical destruction of vertebral body and higher vascularization. Some authors reported the use of a preinjection venogram to decrease the incidence of pulmonary embolism [3, 4].

Conservative management with anticoagulants, antibiotics, and corticosteroids is reserved for smaller or peripherally located cement emboli. Paravertebral venous leakage and pulmonary embolization of cement occurs frequently but is clinically silent in most cases.

In our case, his visual analog scale (VAS) before the procedure was 7 and after the procedure it was 2. In order to prevent or reduce cement leakage, we used high viscosity cement (4 ml volume) and a low-pressure injection. Before injection, we created a cavity with a drill and curette but cement leakages still occurred. It appears that the cement passed from the vertebral venous plexuses via the paravertebral veins.

## Conclusions

The vertebral augmentation with radiofrequency procedure significantly reduced associated spinal malignancy pain, improving functionality and quality of life. However, many patients' cement leakage remains asymptomatic, but late cement migration during follow-up did not occur in our case. Standard post-procedural CT scan of the treated vertebral body in vertebral augmentation is useful. Even though we used high viscosity cement and created a cavity, cement leakage did occur. Close monitoring of patients undergoing VAP and radiological follow-up are important.

## Consent

Written informed consent was obtained from the patient for publication of this case report and any accompanying images. A copy of the written consent is available for review by the Editor-in-Chief of this journal.

## Abbreviations

CT: Computed tomography
MRCL: Myxoid/round cell liposarcoma
MRI: Magnetic resonance imaging
Rf-TA: Radiofrequency thermal ablation
VAPs: Vertebral augmentation procedures
VAS: Visual analog scale
